# Anophelines species and the receptivity and vulnerability to malaria transmission in the Pantanal wetlands, Central Brazil

**DOI:** 10.1590/0074-02760170175

**Published:** 2018-02

**Authors:** Mariana Marinho-e-Silva, Maria Anice Mureb Sallum, Maria Goreti Rosa-Freitas, Ricardo Lourenço-de-Oliveira, Teresa Fernandes Silva-do-Nascimento

**Affiliations:** 1 Fundação Oswaldo Cruz-Fiocruz, Instituto Oswaldo Cruz, Laboratório de Mosquitos Transmissores de Hematozoários, Rio de Janeiro, RJ, Brasil; 2 Universidade de São Paulo, Faculdade de Saúde Pública, Departamento de Epidemiologia, São Paulo, SP, Brasil

**Keywords:** malaria, vectors, wetlands, Pantanal

## Abstract

**BACKGROUND:**

Studies on malaria vectors in the Pantanal biome, Central Brazil, were conducted more than half a century ago.

**OBJECTIVES:**

To update anopheline records and assess receptivity and vulnerability to malaria transmission.

**METHODS:**

Five-day anopheline collections were conducted bimonthly in Salobra, Mato Grosso do Sul state, for one year. Indoors, mosquitoes were collected from their resting places, while in open fields, they were captured using protected human-baited and horse-baited traps near the house and at the Miranda River margin, respectively. Hourly biting activity outdoors was also assessed. Secondary data were collected on the arrival of tourists, economic projects, and malaria cases.

**FINDINGS:**

A total of 24,894 anophelines belonging to 13 species were caught. The main Brazilian malaria vector *Anopheles darlingi* was the predominant species, followed by *An. triannulatus s.l.* Hourly variation in anopheline biting showed three main peaks occurring at sunset, around midnight, and at sunrise, the first and last being the most prominent. The highest density of all species was recorded near the river margin and during the transition period between the rainy and early dry seasons. This coincides with the time of main influx of outsider workers and tourists, whose activities mostly occur in the open fields and frequently start before sunrise and last until sunset. Some of these individuals originate from neighbouring malaria-endemic countries and states, and are likely responsible for the recorded imported and introduced malaria cases.

**MAIN CONCLUSION:**

Pantanal is a malaria-prone area in Brazil. Surveillance and anopheline control measures must be applied to avoid malaria re-emergence in the region.

The main malaria-endemic area in Brazil lies in the Amazon Region; this region accounts for 99% of Brazil's (approximately 125,000) annual malaria cases (MS 2016a, b). However, there are other malaria-prone areas in Brazil outside the Amazon, where all the elements of the malaria transmission cycle are present; these include competent mosquito vectors, naive human populations, malaria-infected humans (commuting and traveling for work and tourism), and favorable environmental conditions. The two main extra-Amazon malaria-prone biomes in Brazil are the coastal Atlantic Forest and the Pantanal Brazilian Central Wetlands (de Pina-Costa et al. 2014), hereinafter called Pantanal (MS 2016a, b). Pantanal occupies 35% of Mato Grosso state (MT) and 65% of Mato Grosso do Sul state (MS) in Brazil. Autochthonous, introduced, and imported malaria cases have been detected annually in the Atlantic Forest and Pantanal biomes (Fig. 1), revealing that these areas are vulnerable and receptive to malaria. Vulnerability is related to human activities such as economic exploitation of natural resources, including cattle breeding, railway construction, hydroelectric plants, mining and tourism activities, as well as other factors that may increase the migration of individuals carrying gametocytes from malaria-endemic areas and/or augment contact with vectors that can trigger the emergence of local malaria outbreaks.

**Fig. 1:**
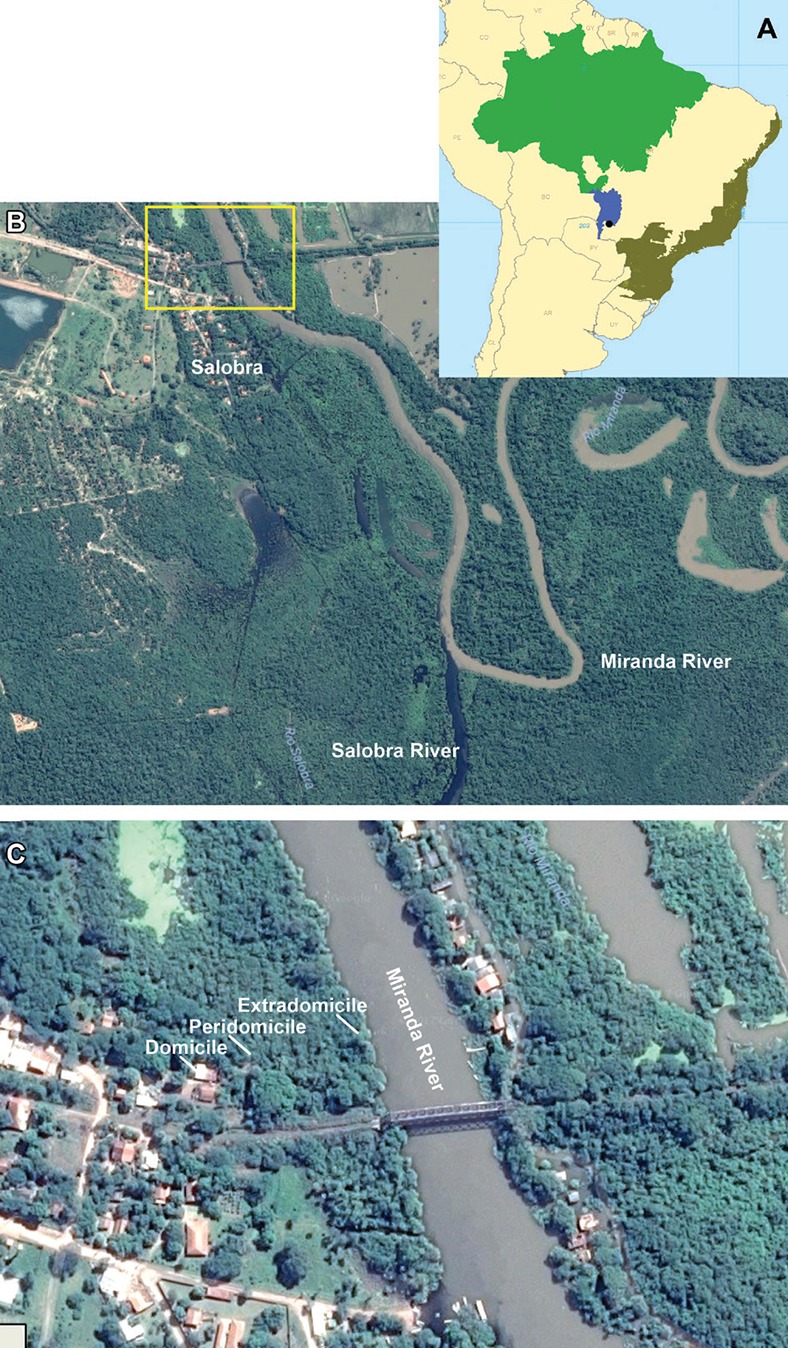
(A) Brazilian biomes: Amazon (green), Pantanal (blue) and Atlantic Forest (brown); (B) Salobra village, Salobra River and Miranda River Basin, Mato Grosso do Sul state, Brazil; (C) collection points for the intradomicile, peridomicile and extradomicile in Salobra next to a Northwest Railroad bridge over the Miranda River.

Source: the authors and Google Earth.

The influx of malaria-infected people from malaria-endemic regions is a key contributing factor to MS and Pantanal's vulnerability. According to the IBGE (Available from: http://www.ibge.gov.br/home/), the Migratory Efficiency Index of MS in 2009 was 0.0712, which is considered high migratory turnover. The main economic activities of MS are agriculture (including livestock farming) and tourism. The tourist attractions in the Pantanal area are seasonal and bring in high revenues.

A region's receptivity to malaria is defined by the presence, density, and longevity of local malaria vectors. Few entomological studies have been conducted in Central Brazil, and rarely in the Pantanal area, over the past decades (Travassos 1939, 1940a, b, Travassos 1941, Travassos & Teixeira de Freitas 1940, 1943). Recent entomological studies have been conducted in areas influenced by the construction of hydroelectric dams to determine the environmental impact caused by dam construction at Indian reservations, outside the Pantanal biome (Ianelli et al. 1998, Gomes et al. 2007, 2010, Ribeiro et al. 2013).

Here, we update the records of anopheline species in Pantanal through a one-year longitudinal entomological study and examine receptivity and vulnerability to malaria.

## MATERIALS AND METHODS

*Locality description and climate data* - The Pantanal biome is administratively occupied by the Brazilian states of MS and MT and extends to the neighbouring countries of Bolivia and Paraguay. Pantanal borders the malarigeneous Brazilian Amazon Region (formed by the Amazon Rain Forest biome) (Fig. 1).

The Pantanal biome is a vast flat marshland of 150,355 km^2^, with extensively flooded areas and lowheight shrubs characteristic of *Cerrado* (Savannah-like vegetation) subject to well-defined periods of flooding and drought. Ecotourism, fishing, hunting, and free-range cattle breeding attract tourists from other parts of Brazil and abroad. There is also a great influx of people into Pantanal for work (GASBOL 2005, Mato Grosso do Sul state government - Available from: http://www.ms.gov.br/institucional/perfil-de-ms).

Anopheline collections were carried out in Salobra (20°12′40″S, 56°29′30″W, 117 m a.s.l.), a village situated at the margin of the Miranda River, in the municipality of Miranda, MS, Brazil (Fig. 1). The collecting sites are located at a distance of approximately 1.5 km from the confluence of the Salobra River and the Miranda River. The term *salobra* means ‘brackish’ in Portuguese and refers to the salinity of the river water (Silva-do-Nascimento & Lourenço-de-Oliveira 2007). The municipality of Miranda is at a distance of approximately 200 km from Campo Grande, the capital of MS (IBGE 2016). Miranda, the “Gateway to the Pantanal”, has a surface area of 5,478 km^2^ and a total population of 25,595 inhabitants (4.67 inhabitants/km^2^). Approximately 37% of Miranda's population lives in rural areas (IBGE - Available from: http://www.ibge.gov.br/home/). The human population of Salobra is mainly concentrated on the banks of the Miranda River (Mendes et al. 2004). The Pantanal climate is classified as subhumid megathermal or Aw (dry winters and rainy summers) according to the Köppen classification. The average annual temperature and relative humidity in this region are 24°C and 72.7%, respectively (SEMAC 2011). In Pantanal, the annual rainfall varies between 1,000 and 1,400 mm (SEMAC 2011) and the period with the highest rainfall is between the months of October and March (Table I). The dry season in Pantanal starts in late May and ends in September; the precipitation can be nil for months (SEMAC 2011). Records of the Miranda River level, rainfall, daily temperature, and other climate data were obtained from the National Water Agency (Agência Nacional de Águas, ANA) and the Ministry of the Environment (stations ANA 66910000 and ANA 02056005).

**TABLE I:** Periods, seasons and lunar phases of mosquito collections conducted in Salobra, Mato Grosso do Sul, Brazil

	Dry season	Rainy season			Dry season	
Date	14-18 Sep/10	08-12 Nov/10	17-21 Jan/11	21-25 Mar/11	23-27 May/11	11-15 Jul/11
Season	Late dry	Transition between dry and rainy	Early rainy	Late rainy	Transition between rainy and dry autumn	Early dry
Lunar phase	Quarter	New moon	Quarter/Full moon	Full moon	Full moon/Waning crescent	Quarter/Full moon
Temperature (Max-Min averages) °C	32-18	29-17	29-22	27-21	27-17	26-14
Rainfall (mm)*^a^*	16.2	126.2	215.2	164.2	0	24.2

a counted from 15 previous days from the collection until the end of the 5-day collections in a total of 20 days. Rainfall and temperature daily records for Salobra showed that the months with the highest precipitation was between January and March 2011 which had 215.2 and 164.2 mm of rain, respectively. The months with the lowest rainfall was September 2010 (late dry season) and May 2011 (end of rainy season).

*Anopheline collections* - Mosquitoes were collected in September and November 2010, and January, March, May, and July 2011 (Table I), for five consecutive days at sunset - from 17:00 h to 20:00 h - simultaneously and comparatively in intradomicile (20°11′51.53″S, 56°30′14.25″W), peridomicile (20°11′51.74″S, 56°30′13.58″W) and extradomicile (20°11′51.21″S, 56°30′10.33″W) areas. The domicile selected for the indoor collections was located at a distance of approximately 100 m from the south margin of the Miranda River (Fig. 1). Inside the domicile, mosquitoes were collected at rest on the internal walls and furniture. Peridomicile collections were performed approximately at a distance of 6 m from the house using a protected human-baited mosquito trap (MosqTent®) (Lima et al. 2017). Extradomicile collections were carried out using a Shannon trap baited with a horse on the south bank of the Miranda River (Fig. 1). The same horse was used every night throughout the study. The Shannon trap and MosqTent® were placed in the same location for all captures.

Mosquitoes were collected using manual Castro aspirators and immediately transferred to cylindrical paper cages. At the end of the collections, most mosquitoes were killed with ethyl acetate and dried on filter paper at room temperature, and a sample of live mosquitoes was brought to the laboratory at Rio de Janeiro.

*Peak of hematophagy* - In order to assess the peak of hematophagy, captures were also carried out for an 18-h period, from 17:00 h to 11:00 h, in each of the months of March, May, and July 2011, using a horse-baited Shannon trap at the same extradomiciliary site described above, with rotation of collectors every three hours.

Mosquito collections were performed under authorisation 64458983 SISBIO/ICMBio/MMA.

*Mosquito species identification* - Mosquitoes were screened by morphological characters of adult females and males, male genitalia (by the shape of the aedeagus and accessory pieces), and larval chaetotaxy. Some of the mosquito samples transferred live to the laboratory were used to obtain offspring for confirmation of species identification using morphological characters (Consoli & Lourenço-de-Oliveira 1994, Forattini 2002, Sallum et al. 2002, Flores-Mendoza et al. 2004, Motoki et al. 2009). Other mosquito samples were killed at -20°C to separate the species belonging to the Triannulatus complex, through biochemical analysis using the isoenzyme mannose phosphate isomerase (MPI), according to the method described by Silva-do-Nascimento et al. (2006). Species identity of specimens belonging to the Oswaldoi and Albitarsis complexes, as well as those morphologically identified as *Anopheles galvaoi* Causey et al., was confirmed by sequencing the mitochondrial gene cytochrome oxidase subunit I-COI (as described in detail in Motoki et al. 2007, Sallum et al. 2010, Ruiz-López et al. 2013) of a sample of dry specimens.

*Anopheline female parity* - Ovaries were dissected in order to determine the parous female rate, which is a proxy for female age (Detinova 1962, Consoli & Lourenço-de-Oliveira 1994). The nulliparous and parous anopheline female rates are used to estimate the portion of the population able to support the full cycle of *Plasmodium* spp. and transmit malaria (Barros et al. 2007). For logistic reasons, female parity was determined in 0.8% and 5.8% of the anophelines collected in May and July 2011 respectively, when the highest anopheline densities were reported.

*Individual human movement* - The annual number of human individuals arriving in Pantanal for work or leisure was obtained from the Mato Grosso do Sul Tourism Foundation (FUNDTUR 2011) (Supplementary data, Table I).

*Malaria cases* - Autochthonous malaria cases reported in MS between 2002 and 2015 were retrieved from the Information System of Reportable Diseases of the Ministry of Health (MS 2016a). Complete records of autochthonous malaria cases that were recorded by MS municipalities between 2007 and 2011 were obtained from the Health State Secretary of MS (unpublished observations). Data were retrieved only up to the year 2011 to coincide with anopheline collections.

## RESULTS

A total of 24,894 anophelines was caught in 330 h (75.4 anophelines per hour) from September 2010 to July 2011 (Table II); among these, 91 were males. Approximately 8% of the mosquito specimens were damaged either during collection or transportation and could not be identified.

**TABLE II:** Total number of anopheline specimens, per species, caught for 3 h at sunset and early night (17:00-20:00 h) and during 18 h collections bimonthly performed from September 2010 to July 2011 in Salobra, Mato Grosso do Sul, Brazil

Species	Sep 2010*^a^*	Nov 2010*^a^*	Jan 2011*^a^*	Mar 2011*^a^*		May 2011*^a^*		Jul 2011*^a^*		Subtotal		Total 3 h+18 h
	3 h	3 h	3 h	3 h	18 h	3 h	18 h	3 h	18 h	3 h	18 h	
*Anopheles darlingi*	75	4	46	1224	201	5321	7522	795	524	7465	8247	15712
*An. albitarsis s.l.* *^b^*	6	8	26	198	61	407	95	234	75	879	231	1110
*An. triannulatus s.l.* *^c^*	202	323	156	908	424	1541	764	502	93	3632	1281	4913
*An. oswaldoi s.l. + An. konderi s.l.*	0	1	71	97	15	118	187	21	15	308	217	525
*An. mattogrossensis*	1	0	16	67	4	73	168	7	2	164	174	338
*An. rondoni*	0	0	2	34	12	82	18	2	0	120	30	150
*An. galvaoi + An. arthuri*	4	7	0	5	3	61	32	36	19	113	54	167
*An. sp*	22	297	10	412	57	390	172	396	223	1527	452	1979
Subtotal 3 h	310	640	327	2945		7993		1993		14208		
Subtotal 18 h					777		8958		951		10686	
Total	310	640	327		3722		16951		2944			24894

a 18 h captures were conducted in March, May and July 2011;

b at least two species belonging to the Albitarsis complex were detected, *An. deaneorum* and *An. oryzalimnetes*;

c *An. triannulatus s.s.*, *An. halophylus* and *An. triannulatus* C.

Morphological and molecular identifications revealed the presence of 13 species or species complexes in Salobra (Tables II–III). Molecular analyses confirmed the identification of eight species. Accordingly, two species belonging to the Albitarsis complex were detected: *Anopheles deaneorum* Rosa-Freitas and *An. oryzalimnetes* Wilkerson and Motoki. Regarding the Oswaldoi complex, only *An. konderi s.l.* Galvão and Damasceno could be confirmed to occur in the area. As expected, the species comprising the Triannulatus complex were detected: *An. triannulatus s.s.* (Neiva & Pinto), *An. halophylus* Silva-do-Nascimento and Lourenço-de-Oliveira (2002), and *An. triannulatus* C Silva-do-Nascimento et al. (2006) (Table III). Finally, analysis of COI sequencing revealed the occurrence of *An. arthuri* Unti and *An. galvaoi*. Since only a few mosquitoes could be analysed by molecular methods, the results concerning species belonging to these complexes are hereafter referred to as *An. albitarsis s.l., An. oswaldoi s.l.* + *An. konderi s.l.*, *An. triannulatus s.l.*, and *An. galvaoi* + *An. arthuri* (Tables III–IV). Voucher specimens were deposited at the Mosquitoes Collection at the Oswaldo Cruz Institute (Coleção de Culicidae, http://cculi.fiocruz.br/).

**TABLE III:** Total number of Triannulatus complex species collected from September 2010 to July 2011 in Salobra, Mato Grosso do Sul, Brazil

Period	*Anopheles triannulatus s.s.*	(%)	*An. halophylus*	(%)	*An. triannulatus C*	(%)	Total (n)
Sept, 2010	35	64.8	17	31.5	2	3.7	54
Nov, 2010	13	24.1	40	74.1	1	1.9	54
Jan, 2011	0	0	15	55.6	12	44.4	27
Mar, 2011	35	85.4	5	12.2	1	2.4	41
May, 2011	16	37.9	11	37.9	2	6.9	29
Jul, 2011	11	82.8	82	82.8	6	6.1	99
Total	110		170		24		304 (100%)

**TABLE IV:** Anopheline species collected in the intradomicile, in the peridomicile (protected human-baited) and in the extradomicile (horse-baited Shannon trap) from September 2010 to July 2011 in Salobra, Mato Grosso do Sul, Brazil*^a^*

Species	Intra-domicile	(%)	Peri-domicile	(%)	Extra-domicile	(%)	Total	(%)
*Anopheles darlingi*	12	0.10	84	0.68	7221	58.31	7317	59
*An. albitarsis s.l.* *^b^*	26	0.21	69	0.56	778	6.28	873	7
*An. triannulatus s.l.* *^c^*	24	0.19	70	0.57	3442	27.80	3536	29
*An. oswaldoi s.l.* + *An. konderi s.l.*	5	0.04	30	0.24	241	1.95	276	2
*An. mattogrossensis*	4	0.03	11	0.09	139	1.12	154	1
*An. rondoni*	0	0.00	3	0.02	112	0.90	115	1
*An. galvaoi* + *An. arthuri*	2	0.02	3	0.02	107	0.86	112	1
Total	73	1	270	2	12040	97	12383	100

a damaged non-identifiable caught specimens were not considered;

b at least two species belonging to the Albitarsis complex were detected, *An. deaneorum* and *An. oryzalimnetes*;

c *An. triannulatus s.s.*, *An. halophylus* and *An. triannulatus* C.

The most frequent species or species complexes were *An. darlingi* Root and *An. triannulatus s.l.*, which together constituted 82.9% of the total specimens captured in Salobra. Species captures at lower frequencies were *An. albitarsis s.l.* (4.5%), *An. oswaldoi s.l.* + *An. konderi s.l.* (2.1%), *An. mattogrossensis* Lutz and Neiva (1.4%), *An. galvaoi* + *An. arthuri* (0.7%), and *An. rondoni* (Neiva & Pinto) (0.6%).

**Fig. 2:**
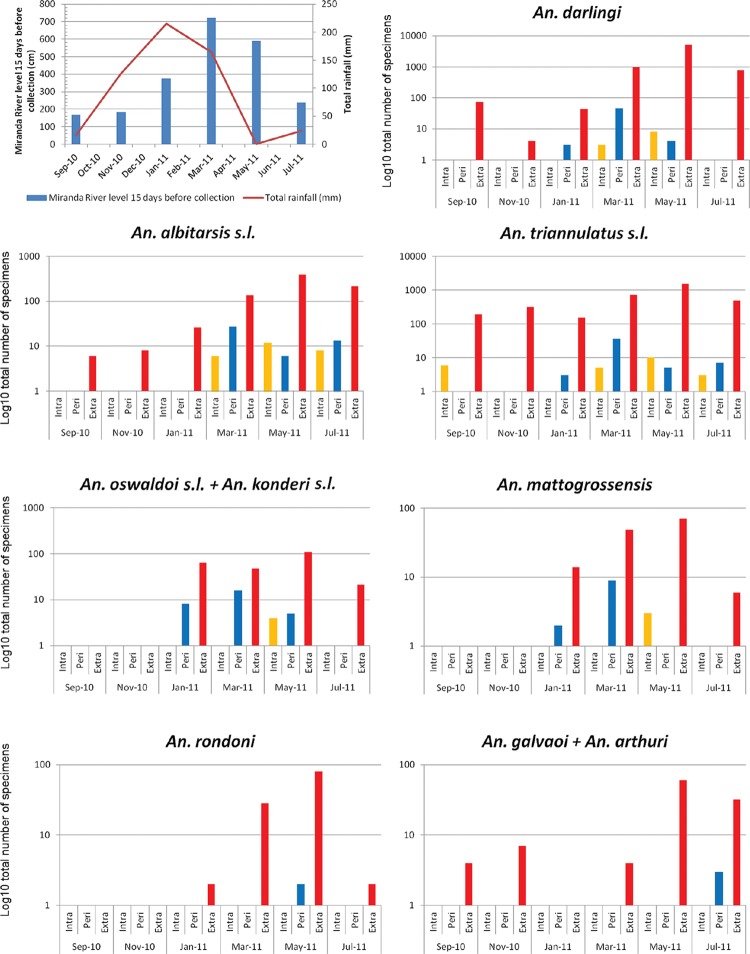
total number of anopheline specimens (y-axis log_10_) per species, captured at sunset from September 2010 to July 2011 indoors (yellow) and in protected human-baited (blue) and horse-baited (red) traps settled at the peri- and extradomicile collecting sites, respectively, and rainfall and water level of the Miranda River in Salobra, Mato Grosso do Sul, Brazil.

All anopheline species were more frequently collected during the transition between rainy and dry seasons, which occurs in May, the month in which no rain is recorded (Tables II–III, Fig. 2). This transition coincides with the beginning of the reduction in the water level of the main rivers (Miranda and Salobra) and the establishment of large and stable water bodies (Fig. 2). *An. darlingi* was the predominant species from the late rainy to early dry season, while *An. triannulatus s.l.* was the prevalent anopheline from the late dry season to the beginning of the rainy period (Tables II–III). Concerning the latter species complex, molecular screening of bimonthly samplings suggested that *An. halophylus* was the predominant member from the early dry to the early rainy season (Table III).

All anopheline species exhibited exophagic and zoophagic feeding behaviour, as the great majority of mosquitoes were captured in the horse-baited Shannon trap at the riverbank regardless of the season (Fig. 2). However, all species were also attracted to humans, as revealed by the captures both inside and outside the house, especially captures of *An. darlingi*.

Among the 12,283 anophelines caught in the sunset collections, the largest portion was caught extradomicile (98%, or 12,020 specimens), 60% of which were *An. darlingi*, followed by *An. triannulatus s.l.* at 28% (Table IV). Only 0.5% and 1.5% of the anophelines were caught indoors and in the protected human-baited trap at peridomicile, respectively. The species most frequently found indoors was *An. albitarsis s.l.* (26 out of 73 specimens, or 36%), while the most frequent species at peridomicile were *An. darlingi* and *An. triannulatus s.l.* (28%, or 53 out of 190 specimens, each).

**Fig. 3:**
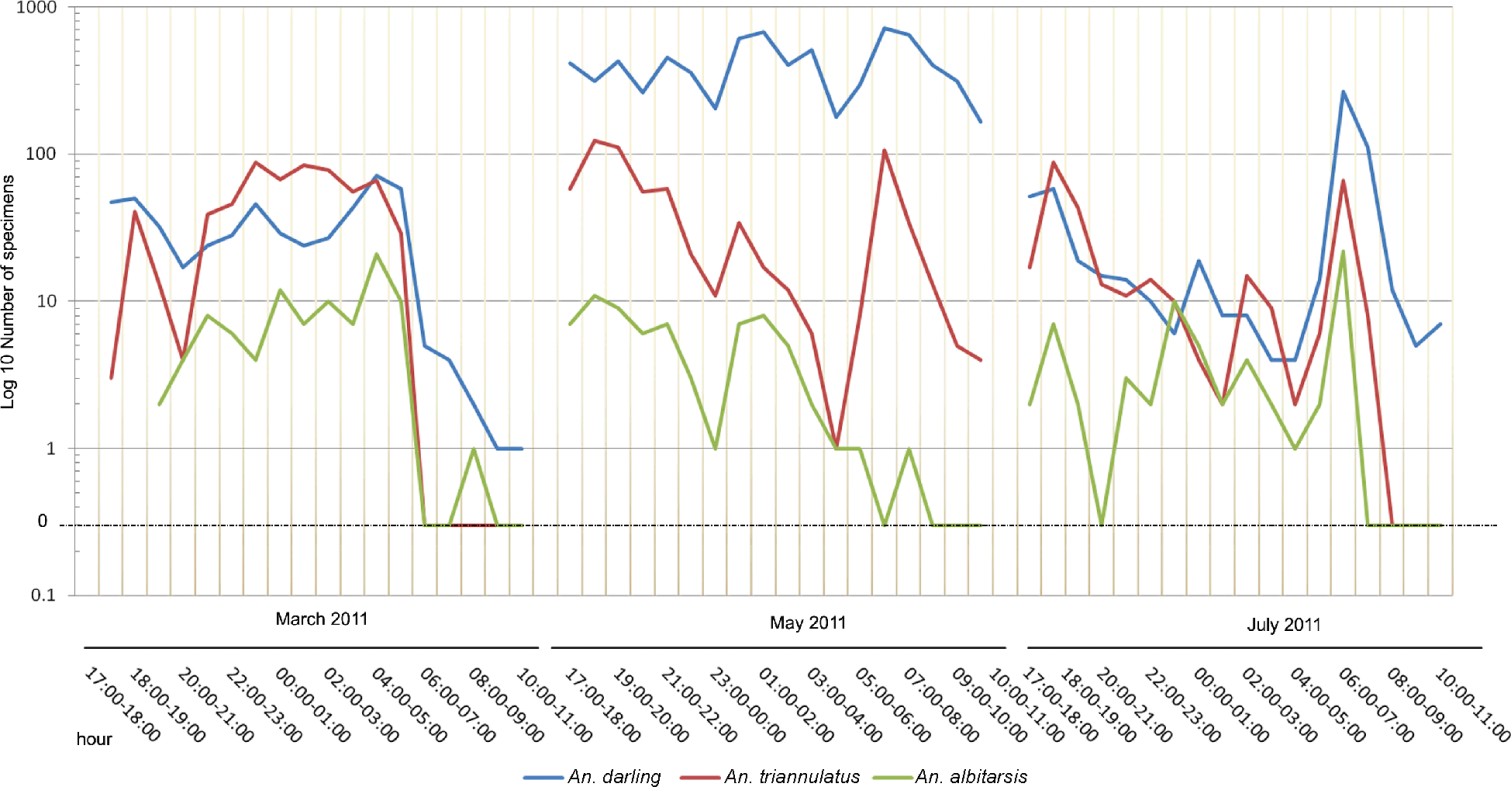
*Anopheles darlingi*, *An. triannulatus s.l.*, and *An. albitarsis* specimens caught in 18-h collections carried out in March, May, and July 2011 in Salobra, Mato Grosso do Sul state, Brazil.

A total of 10,686 anophelines was caught in the three 18-h collections performed to characterise the biting peak in March, May, and July 2011. Hourly variation in anopheline collections essentially showed three main peaks of hematophagy, occurring at sunset, around midnight, and at sunrise (Fig. 3). However, the three most abundant species - *An. darlingi*, *An. triannulatus s.l.*, and *An. albitarsis s.l.* - displayed some differences in their peaks of hematophagic activity among species as well as according to the season. For some anopheline species, the number of specimens captured did not exceed 25, making it difficult to assess their peaks of hematophagic activity.

Ovaries of a total of 309 anophelines collected extradomicile were dissected in May 2011 (n = 136, or 0.8% of the total collected) and July 2011 (n = 173, or 5.8% of the total collected). The proportions of parous females of *An. darlingi* and *An. triannulatus s.l.* in these two samplings varied from 38 to 40% and 33 to 60.2%, respectively.

Tourism is currently responsible for one of the largest movements of people in the Pantanal area (Supplementary data, Table I). In 2010, almost 70,000 tourists visited Brazil, with MS being the entryway (FUNDTUR 2011). The influx of people into the state has increased annually, especially via air transportation, with 96% of arrivals originating from neighboring South American malaria-endemic countries such as Bolivia and Paraguay (FUNDTUR 2011). Farming, tourism, and other economic activities are seasonal in Pantanal due to the flooding that peaks from the late rainy to the early dry season. A gas pipeline project that crosses the state as far as Bolivia has also brought in workers, mainly from this country (GASBOL 2005). Extensive flooding during the rainy season and the resilient drought in the late dry season strongly impair locomotion, livestock, tourism, and most economic activities in the open fields in Pantanal. Regarding malaria, MS recorded 472 cases from 2002 to 2015 (Supplementary data, Table II), 39 of which were autochthonous or introduced cases in or near Miranda, the municipality where Salobra is located; 433 were imported malaria cases (mainly from the Amazonian states of Rondônia, Mato Grosso, and Pará) (MS 2016a, b). Most infections (75%) were due to *Plasmodium vivax*, of which 67% occurred in males aged 18 to 52 years (Marinho-e-Silva 2012).

## DISCUSSION

The Salobra area, one of the main gateways to the Brazilian Pantanal marshlands, has a rich anopheline fauna composed of several malaria vector species, notably *An. darlingi*, as well as species belonging to the Albitarsis, Triannulatus, and Oswaldoi complexes (Rosa-Freitas et al. 1998, Póvoa et al. 2001, Silva-do-Nascimento & Lourenço-de-Oliveira 2007, Motoki et al. 2009).

Indeed, the distinct water characteristics (fresh versus brackish water), the landscape and the dry/rain regimen result in flooding of vast lowlands at the riverbanks, creating abundant and diverse breeding sites for mosquitoes, especially anophelines. This condition favours the diversity of anopheline fauna, as revealed by the detection of 13 species in this study; among these, *An. arthuri*, *An. konderi s.l.*, *An. oryzalimnetes*, *An. deaneorum*, and *An. galvaoi* are described for the first time in this part of Pantanal; the last three species are newly recorded in Mato Grosso do Sul state.

Anopheline density in Pantanal is strongly influenced by the water level of the main rivers and flooding of adjacent lowlands. During the rainy season, with river overflow and rapid flooding, immature stages of anophelines are washed from unstable breeding sites; thus, anopheline adult density decreases. On the other hand, during the transition period between the rainy and the dry seasons and at the beginning of the dry season, when water impoundments receive little or no inflow, anopheline density increases in the area (Silva-do-Nascimento & Lourenço-de-Oliveira 2007). This was clearly the case for *An. darlingi*. Coincidently, the number of tourists/visitors and outsider workers attracted by temporary farming and other activities peaks in the region in this period (FUNDTUR 2011). The parity data of *An. triannulatus s.l.* in Brazil had never been recorded in the literature. Our data showed that 60% and 40% of *An. triannulatus s.l.* and *An. darlingi*, respectively, were parous in the early dry season. Taken together, these data suggest that this time of year has the highest risk of malaria transmission in the Pantanal.

All anopheline species, most markedly *An. darlingi*, were much more frequent outdoors. Moreover, *An. darlingi* was shown to be active all night long in this environment, with the highest biting peak around sunrise, but also biting in the day time (at least until 11:00 h) in the transition between the rainy and dry seasons (May) as well in the early dry season (July). This biting pattern was previously observed elsewhere (Charlwood & Hayes 1978, Tadei et al. 1988, 1998, Lourenço-de-Oliveira et al. 1989, Klein & Lima 1990, Rosa-Freitas et al. 1992). Notably, the abovementioned seasonal human activities (e.g. ecotourism, sport fishing, hunting, and free-range cattle breeding) are mostly performed in open fields and may start before sunrise and last until sunset, favouring contact between humans and exophagic vectors, and thus triggering local malaria transmission. The predominance of the 2007-2011 malaria cases in MS being in males aged 18 to 52 years was related to professional activities, which suggests that the infections may have been acquired outdoors (Marinho-e-Silva 2012). This pattern is in accordance with that in other extra-Amazon regions in Brazil, where approximately 70% of the individuals who have malaria are males (Miguel et al. 2014, de Pina-Costa et al. 2014).

Besides *An. darlingi*, considered the main malaria vector in Brazil, other anophelines such as *An*. *triannulatus s.l.* and *An. albitarsis s.l.* were observed at relatively high frequencies in Salobra. These species are considered putative secondary vectors and can sustain malaria transmission, especially when present at high densities (Deane et al. 1948, Arruda et al. 1986, Tadei & Thatcher 2000, Póvoa et al. 2001).

In conclusion, in Pantanal, a malaria-prone area in Brazil, the high density of anophelines that are competent malaria vectors combined with the seasonal influx and movement of people increase the possibility of malaria-infected individuals moving from malaria-endemic regions to this receptive area and reinforce the vulnerability of this large area to malaria transmission. Continuous epidemiological surveillance should be carried out to prevent the recurrence of malaria outbreaks and the reestablishment of endemic malaria in Pantanal.
