# Supplementary material for: Anophelines species and the receptivity and vulnerability to malaria transmission in the Pantanal wetlands, Central Brazil
Source: Mem Inst Oswaldo Cruz. 2018 Feb;113(2):87–95. doi: 10.1590/0074-02760170175 (PMC5722263; doi:10.1590/0074-02760170175)
Supplement: Supplementary file 1 [file 0074-0276-mioc-113-02-0087-Suppl01.pdf]

TABLE I

Annual land arrivals by country (top 20 countries of origin) in Mato Grosso do Sul state, Brazil, during the years 2007-2015

Country	2007	2008	2009	2010	2011	2012	2013	2014	2015
Bolivia	22,566	24,642	31,938	37,376	20,333	29,132	27,504	27,840	28,135
Paraguay[a,b]	19,879	19,382	21,411	23,169	14,663	8,373	7,978	23,304	21,264
Peru	4,674	1,622	3,413	4,136	1,068	1,568	1,663	2,660	1,582
England[a]	1,083	441	117	419	351	525	478	544	300
Germany[a,b]	807	212	118	271	231	363	351	497	398
Australia	695	490	128	295	211	267	213	294	125
USA[a]	627	8	10	4	94	159	118	194	195
Argentina[a,b]	633	327	262	329	389	503	472	1,129	1,270
Spain[b]	619	149	92	71	133	142	138	196	182
Canada[b]	371	-106	43	104	62	102	90	100	101
France[a]	368	235	54	180	188	362	319	400	393
Netherlands[b]	341	141	47	111	91	164	159	212	110
Switzerland[b]	165	119	54	110	69	120	105	126	126
Chile[a,b]	122	229	151	214	128	298	419	1,708	397
Colombia	87	84	97	197	118	227	212	872	337
Equator	123	90	38	70	146	286	160	414	172
Uruguay[a,b]	75	32	25	53	35	50	24	45	44
Italy[a,b]	75	83	27	58	47	82	103	114	95
Mexico	-		-	-	-	36	110	352	379
Portugal[a,b]	31	17	*	12	32	38	52	66	64

a: top 10 countries with tourist arrivals in Brazil in 2015; *b*: top 12 countries for which leisure for nature, ecotourism, and adventure represented > 53% of the reason for traveling to Brazil in 2015 (FUNDTUR 2015).

TABLE II

Number of malaria cases reported in Mato Grosso do Sul state, from 2002 to 2015[a]

Year	Autochthonous	Imported	Total cases
2002	11	31	42
2003	10	44	54
2004	4	54	58
2005	3	70	73
2006	0	61	61
2007	1	26	27
2008	0	19	19
2009	4	29	33
2010	1	21	22
2011	3	21	25
2012	2	24	26
2013	0	19	19
2014	0	8	8
2015	0	6	6
Total	39	433	472

a: SINAN Web/SVS/MS (MS 2016a, b).
